# Identifying the receptor subtype selectivity of retinoid X and retinoic acid receptors via quantum mechanics

**DOI:** 10.1002/2211-5463.12188

**Published:** 2017-02-05

**Authors:** Motonori Tsuji, Koichi Shudo, Hiroyuki Kagechika

**Affiliations:** ^1^ Institute of Molecular FunctionSaitamaJapan; ^2^ Japan Pharmaceutical Information CenterShibuya‐kuTokyoJapan; ^3^ Institute of Biomaterials and BioengineeringTokyo Medical and Dental UniversityChiyoda‐kuJapan

**Keywords:** drug design, quantum mechanics, receptor subtype selectivity, retinoic acid receptors, retinoid X receptors, retinoids

## Abstract

Understanding and identifying the receptor subtype selectivity of a ligand is an important issue in the field of drug discovery. Using a combination of classical molecular mechanics and quantum mechanical calculations, this report assesses the receptor subtype selectivity for the human retinoid X receptor (hRXR) and retinoic acid receptor (hRAR) ligand‐binding domains (LBDs) complexed with retinoid ligands. The calculated energies show good correlation with the experimentally reported binding affinities. The technique proposed here is a promising method as it reveals the origin of the receptor subtype selectivity of selective ligands.

**Abbreviations**
9cRA: 9‐*cis* retinoic acid
ATRA: all‐*trans* retinoic acid
FMO: fragment molecular orbital
IE: interaction energy
IFIE: interfragment interaction energy
LBD: ligand‐binding domain
MP2: second‐order Møller–Plesset perturbation
ONIOM: our own n‐layered integrated molecular orbital and molecular mechanics
RAR: retinoic acid receptor
RXR: retinoid X receptor
SCF: self‐consistent field

Retinoid X receptors (RXRs) and retinoic acid receptors (RARs) are class 1 and class 2 nuclear receptors (NRs), respectively 1. Both receptors exhibit α, β, and γ subtypes. RXRs and RARs form heterodimers, which control crucial biological events such as cell differentiation and proliferation, morphogenesis, and homeostasis. All‐*trans* retinoic acid (ATRA; Fig. 1) is a natural ligand of RARs. Its geometrical isomer, 9‐*cis* retinoic acid (9cRA; Fig. 1), is thought to be a natural ligand of RXRs, although 9cRA also strongly binds to RARs. Recently, molecular mechanics docking simulations were used to investigate the binding of ATRA to RXRs, and this study suggested that ATRA could act as an endogenous ligand of RXRs 2. Moreover, the helix H3 three‐point initial‐binding hypothesis of ligand in the ligand‐binding domains (LBDs) of the NR superfamily was proposed, and the driving forces behind ligand entry into the NR LBDs were discovered, leading to the successful use of molecular dynamics (MD) simulations to understand the structural transition between the apo‐form and holo‐form of both hRXRα and hRARγ LBDs 3. Furthermore, the local motifs that determine the canonical fold of NR LBDs were discovered, and the agonism and antagonism of the NR superfamily at the electron level were proposed 1.

**Figure 1:**
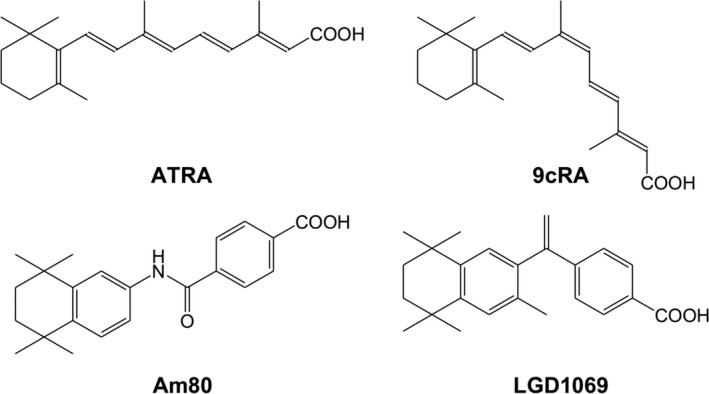
Naturally occurring (ATRA and 9cRA) and synthetic retinoids (Am80 and LGD1069).

In this study, the origin of receptor subtype ligand selectivity was investigated for hRXRs and hRARs with their natural ligands (9cRA and ATRA; Fig. 1) and the synthetic ligands LGD1069 4 (Bexarotene) and Am80 5 (Tamibarotene) (Fig. 1). LGD1069 is a representative RXR‐selective ligand and Am80 is a representative RAR‐selective ligand. Although some studies analyzed the binding affinities of different compounds for only one receptor by using quantum mechanics calculations, investigation of the correlation among receptor subtypes with different compounds has thus far not been reported in the literature. Here, a promising method for understanding the origin of receptor subtype selectivity with these selective ligands is demonstrated. Specifically, second‐order Møller–Plesset perturbation (MP2) theory calculations, in conjunction with ONIOM method 6, were undertaken to evaluate the binding free energies (Δ*G* ^bind^) between the receptors and ligands. In addition, MP2 theory calculations using the fragment molecular orbital (FMO) method 7 were performed on the entire receptor–ligand system to analyze the interaction energies between the receptors and ligands.

## Materials and methods

With the exception of the hRXRγ LBD, the three‐dimensional (3D) structures of the liganded LBDs of hRXRs and hRARs in the agonist conformation have been obtained using X‐ray crystal structure analysis. In this study, the initial structures were prepared using Homology Modeling Professional for hyperchem 8, 9 (each structure contains a coactivator fragment and water molecules), as described in a previous report 2. The 3D structure of hRXRγ LBD in the agonist conformation was prepared using a homology modeling technique, as previously described 2. Docking simulations (AMBER99 force field) using Docking Study with hyperchem 9, 10 were performed under biomacromolecule‐rigid and ligand‐flexible conditions, as previously described 2. With the exception of the water molecules conserved in the ligand‐binding site, water molecules as well as the coactivator fragment were removed during this stage. A Gaussian 11 job file for the most stable complex was automatically prepared using ONIOM Interface for Receptor 12 integrated into the Homology Modeling Professional for hyperchem software. The three‐layer ONIOM calculations (B3LYP/6‐31G*:AM1:AMBER) were carried out for all combinations of LBDs with the ligands; the ligand was defined as the high layer (B3LYP/6‐31G*), the amino acid residues and conserved water molecules positioned within 4 Å of the heavy atoms of the ligand were defined as the medium layer (AM1), and the remaining structures were defined as the low layer (AMBER). In these calculations, the structures of both high and medium layers were fully optimized, whereas only hydrogen atom positions were optimized for the low layer. The binding free energies (Δ*G* ^bind^) in the gas phase at 298.15 K were obtained from the single‐point frequency analysis for the converged complex (using two‐layer ONIOM calculations; MP2/6‐31G:AMBER) and the isolated receptor (AMBER) and ligand (MP2/6‐31G). For the converged structures, FMO calculations were performed at the MP2 level of theory using the ABINIT‐MP program 13. The job files were prepared using BioStation Viewer 14. Interfragment interaction energies (IFIE) for the ligands were obtained using single‐point calculations at the MP2/6‐31G level of theory.

## Results and Discussion

**Figure 2:**
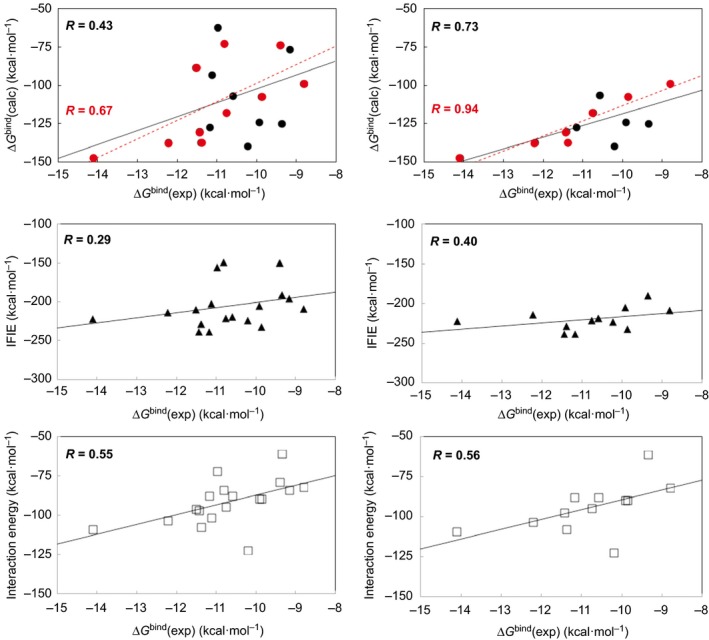
Correlations between Δ*G* ^bind^(exp) 15 and Δ*G* ^bind^(calc) (circles), IFIE (triangles), and interaction energies of the most stable complex obtained from the docking simulations (squares) for the binding of the α, β, and γ subtypes of hRXR and hRAR LBDs with ATRA, 9cRA, Am80, and LGD1069. Left‐hand side shows the correlation diagram for all six receptors. Right‐hand side shows the correlation diagram for hRXRα, hRXRβ, hRARβ, and hRARγ LBDs. At the top, red color represents the correlation for ATRA and 9cRA.

It has recently been reported that the interaction energies obtained from biomacromolecule‐rigid and ligand‐flexible docking simulations using a classical molecular mechanics force field showed excellent correlations with the experimental binding affinities of 9cRA and ATRA 2. The docking simulations for structurally different synthetic ligands have now been investigated under the same conditions (Table 1 and Fig. 2). Since the RXR subtype‐selective ligand is not obtained experimentally, LGD1069 was chosen in this study. On the other hand, Am80 exhibits RARα and RARβ subtype selectivity. From the results, it is apparent that the correlations between the calculated interaction energies and experimental binding affinities are not as strong as previously reported, although there is still some level of agreement (Fig. 2; bottom). It seems that the structure of the ligand‐binding site is strongly dependent on the crystal structure with the natural ligands, that is, 9cRA and ATRA. Biomacromolecule‐ and ligand‐flexible docking simulations were also performed but the correlations between the calculated interaction energies and experimental binding affinities were not improved (see Table S1 and Fig. S1).

**Table 1:** Experimental Δ*G* ^bind^ values 15 and calculated energies of naturally occurring and synthetic retinoids

Receptor	Ligand	Δ*G* ^bind^(exp)^a^(kcal·mol^−1^)	ONIOM (MP2/6‐31G: AMBER) Δ*G* ^bind^ (calc) (kcal·mol^−1^)	FMO (MP2/6‐31G) IFIE (kcal·mol^−1^)	Docking IE (kcal·mol^−1^)
hRXRα (PDB ID: 1FM9)	ATRA	−8.81	−98.70	−208.34	−81.83
	9cRA	−10.76	−117.92	−221.14	−94.41
	Am80	nb	–	–	nh
	LGD1069	−10.59	−106.70	−218.55	−87.55
hRXRβ (PDB ID: 1UHL)	ATRA	−9.87	−107.35	−231.73	−89.36
	9cRA	−11.44	−130.52	−238.15	−97.08
	Am80	nb	–	–	nh
	LGD1069	−11.18	−127.32	−238.15	−87.77
hRXRγ (Model)	ATRA	−9.40	−73.75	−149.30	−78.60
	9cRA	−10.81	−72.57	−149.11	−83.76
	Am80	nb	–	–	nh
	LGD1069	−10.98	−61.86	−154.81	−72.02
hRARα (PDB ID: 3A9E)	ATRA	−11.52	−88.37	−209.22	−95.94
	9cRA	nd	−84.31	−198.00	−94.03
	Am80	−11.12	−92.90	−202.27	−101.63
	LGD1069	−9.16	−76.64	−195.29	−84.03
hRARβ (PDB ID: 4DM8)	ATRA	−11.39	−137.46	−228.61	−107.72
	9cRA	nd	−131.73	−223.41	−112.03
	Am80	−10.22	−139.99	−223.06	−122.27
	LGD1069	−9.92	−124.27	−204.95	−89.47
hRARγ (PDB ID: 2LBD)	ATRA	−14.13	−147.43	−221.82	−108.99
	9cRA	−12.23	−137.60	−213.63	−103.19
	Am80	nb	–	–	nh
	LGD1069	−9.35	−124.97	−190.48	−60.91

nb, does not bind; nd, no data available; nh, no hit.

a Ref. 15.

The structures of the ligand‐binding site and complexed ligand obtained from the docking simulations were further optimized using QM/MM ONIOM calculations at the B3LYP/6‐31G*:AM1:AMBER scheme. For the optimized structures, the single‐point two‐layer ONIOM calculations (MP2/6‐31G:AMBER) for the complexes and the single‐point calculations of the isolated receptors (AMBER) and ligands (MP2/6‐31G) were performed to obtain the Δ*G* ^bind^(calc) values. The Δ*G* ^bind^(calc) values in the gas phase showed good correlation, with a correlation coefficient (R) of 0.43 (Fig. 2; top left). As is apparent from the right‐hand side of Fig. 2 and from Table 1, with the exception of hRXRγ and hRARα LBDs, the correlation coefficient for the LBDs was high (0.73). This underlines the suitability of the method used in this study (when only 9cRA and ATRA were plotted for the four receptors, R was 0.94, thus strengthening our previous conclusions 2). Only the 3D structure of hRXRγ LBD was prepared by homology modeling, and this may be a reason for the low correlation observed for hRXRγ LBD; however, the reason for the low correlation for hRARα LBD is unclear (it may be attributed to allosteric effect from the antagonistic heterodimer partner of the original structure, as shown in Table S2).

Single‐point calculations for the whole system were then performed using the FMO method at the MP2/6‐31G level of theory. Under these conditions, all SCF calculations converged and the resulting IFIE values were reasonable. The middle of Fig. 2 shows the correlations between the obtained IFIE values and experimental Δ*G* ^bind^ values for the six receptor subtypes studied. The IFIE values showed poor correlation, with a correlation coefficient of 0.29 (Fig. 2; middle left). However, as is apparent from Table 1, with the exception of hRXRγ LBD, the correlation coefficient for the LBDs improved to 0.41 (at the middle right of Fig. 2, R was 0.40 for the four receptors, with the exception of hRXRγ and hRARα LBDs). It seems that the geometrical optimization calculations at the MP2 level of theory with larger basis set will be needed for the FMO method for the whole system.

**Figure 3:**
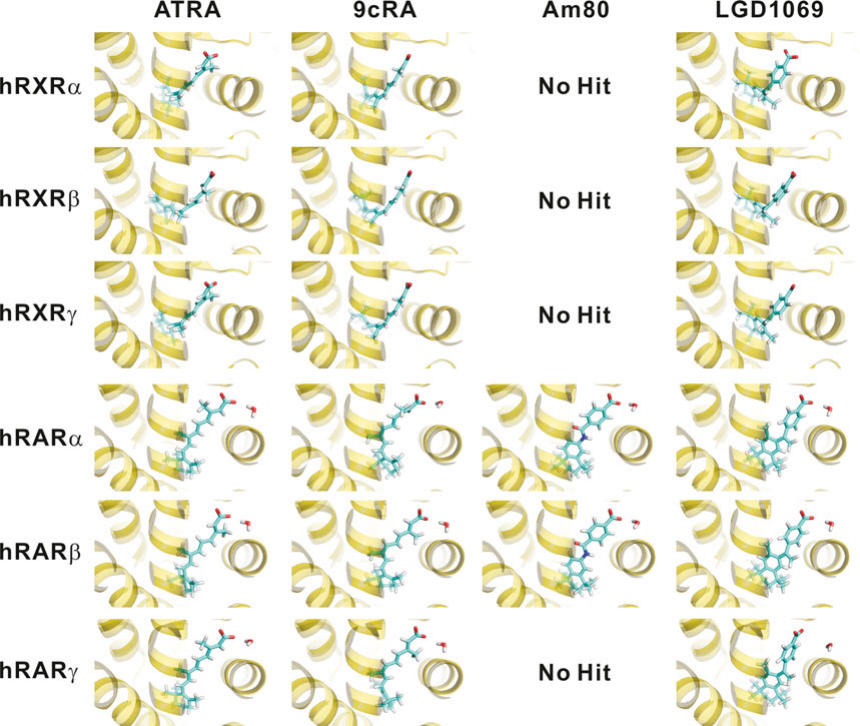
Three‐layer ONIOM (B3LYP/6‐31G*:AM1:AMBER)‐optimized structures of the most stable complex obtained from the biomacromolecule‐rigid and ligand‐flexible docking simulations for the binding of the α, β, and γ subtypes of hRXR and hRAR LBDs with ATRA, 9cRA, Am80, and LGD1069. The structures of ATRA and 9cRA were modified (or further optimized) from the previous structures 2, although these docking poses were maintained.

Table 2 shows the amino acid residues of the ligand‐binding sites of hRXRs and hRARs. The residues involved in hRXR ligand‐binding sites are identical regardless of subtype, underscoring the difficulty faced during the assessment of receptor subtype selectivity. In contrast, one (α and β), two (β and γ), or three (α and γ) residue(s) of the ligand‐binding sites of hRARs differ(s) from each other. The docking simulations support the experimental observation that Am80 does not bind to hRARγ as well as hRXRs (Table 1) and suggest that Am80 is able to recognize the structure of the ligand‐binding site. Although the sequences of RXR and RAR LBDs are moderately conserved, with approximately 30% identity, 9cRA and ATRA can bind to both receptors. For example, there are only three identical ligand‐binding residues in the LBDs of hRXRα and hRARα: Leu309 and Leu269, Ile310 and Ile270, and Arg316 and Arg276 (Table 2). The ability of ATRA and 9cRA to bind to both receptors despite the different residues in the binding sites could be attributed to the conformational flexibility of these naturally occurring retinoids 2, which is absent in the synthetic retinoids (Fig. 3). As shown in Fig. 3, the structure of LGD1069 is more flexible than that of Am80; thus, LGD1069 can bind to both hRXR and hRAR LBDs, and its agonistic potency for hRXRs is in the range of at most one or two order(s) of magnitude greater than that for hRARs 15. Therefore, the receptor subtype selectivity of the ligands could not be accounted for by considering only the differences in the binding residues. Rather, it appears that the binding affinities of the ligands can instead be estimated from the Δ*G* ^bind^(calc) values obtained from quantum mechanical MP2 calculations for the entire receptor–ligand system.

**Table 2:** Amino acid residues of the ligand‐binding sites of hRXRs and hRARs

hRXRα	hRXRβ	hRXRγ	hRARα	hRARβ	hRARγ
			F199	F199	F201
I268	I339	I269	F228	F228	F230
A271	A342	A272	L231	L231	L233
A272	A343	A273	**S232**	**A232**	**A234**
Q275	Q346	Q276	C235	C235	C237
N306	N377	N307	L266	L266	L268
L309	L380	L310	L269	L269	L271
I310	I381	I311	**I270**	**I270**	**M272**
S312	S383	S313	R272	R272	R274
F313	F384	F314	I273	I273	I275
R316	R387	R317	R276	R276	R278
L326	L397	L327	F286	F286	F288
A327	A398	A328	S287	S287	S289
V342	V413	V343	F302	F302	F304
I345	I416	I346	L305	L305	L307
C432	C503	C433	G391	G391	G393
H435	H506	H436	R394	R394	R396
L436	L507	L437	**V395**	**V395**	**A397**
F439	F510	F440	L398	L398	L400
			M413	M413	M415
			L414	L414	L416

Residues that differ between the subtypes are represented in bold face. The sequence alignments of hRXR and hRAR LBDs have been reported in Ref. 1.

## Conclusion

This study demonstrates that receptor subtype selectivity can be predicted from energies calculated for an entire receptor–ligand system using MP2 quantum mechanical calculations. Quantum mechanical geometrical optimization of the complexed ligand and the surrounding region was essential for obtaining reliable values. The conventional method, wherein the initial structure is prepared using classical molecular mechanics calculations such as geometry optimizations and MD equilibrations, was insufficient to evaluate the binding affinities.

In conclusion, the method described in this study proved useful for assessing the receptor subtype selectivity of ligands. The origin of the receptor subtype selectivity of ligands highlighted by these results remains the subject of further investigation.

## Author contributions

MT, KS, and HK designed the experiments. MT performed the experiments, analyzed the data, and wrote the paper. All authors approved the manuscript.

## Supporting information


**Table S1.** Experimental Δ*G* ^bind^ values and calculated interaction energies of naturally occurring and synthetic retinoids.
**Table S2.** Receptor information used in this study.
**Fig. S1.** Correlations between Δ*G* ^bind^(exp) and interaction energies of the most stable complex obtained from the docking simulations (the biomacromolecule‐ and ligand‐flexible conditions) for the binding of the α, β, and γ subtypes of hRXR and hRAR LBDs with ATRA, 9cRA, Am80, and LGD1069.
