# Supplementary material for: Identifying the receptor subtype selectivity of retinoid X and retinoic acid receptors via quantum mechanics
Source: FEBS Open Bio. 2017 Feb 5;7(3):391–6. doi: 10.1002/2211-5463.12188 (PMC5337894; doi:10.1002/2211-5463.12188)
Supplement: Supplementary file 1 — Table S1. Experimental ΔG bind values and calculated interaction energies of naturally occurring and synthetic retinoids. Table S2. Receptor information used in this study. Fig. S1. Correlations between ΔG bind(exp) and interaction energies of the most stable complex obtained from the docking simulations (the biomacromolecule‐ and ligand‐flexible conditions) for the binding of the α, β, and γ subtypes of hRXR and hRAR LBDs with ATRA, 9cRA, Am80, and LGD1069. [file FEB4-7-391-s001.pdf]

Supporting Information

Identifying the receptor subtype selectivity of retinoid X and retinoic acid receptors via quantum mechanics

Motonori Tsuji,*[,1] Koichi Shudo,[2] and Hiroyuki Kagechika[3]

[1] Institute of Molecular Function, 2-105-14 Takasu, Misato-shi, Saitama 341-0037, Japan

[2] Japan Pharmaceutical Information Center, 2-12-15 Shibuya, Shibuya-ku, Tokyo 150-0002, Japan

[3] Institute of Biomaterials and Bioengineering, Tokyo Medical and Dental University, 2-3-10 Kanda-Surugadai, Chiyoda-ku, Tokyo 101-0062, Japan

E-mail: motonori@molfunction.com

Contents

The correlations between ΔG^{bind}(exp) and interaction energies of the most stable complex obtained from the biomacromolecule- and ligand-flexible docking simulations. **S2-S3**

Receptor information used in this study. **S3**

References **S4**

Table S1. Experimental ΔG^{bind} values [1] and calculated interaction energies of naturally occurring and synthetic retinoids.

Receptor	Ligand	ΔG^{bind}(exp)[a] (kcal/mol)	Docking IE (kcal/mol)	Flexible Docking IE (kcal/mol)
hRXRα (PDB ID: 1FM9)	ATRA	-8.81	-81.83	-96.28
	9cRA	-10.76	-94.41	-98.00
	Am80	nb[b]	nh[d]	nh[d]
	LGD1069	-10.59	-87.55	-98.13
hRXRβ (PDB ID: 1UHL)	ATRA	-9.87	-89.36	-102.94
	9cRA	-11.44	-97.08	-104.40
	Am80	nb[b]	nh[d]	nh[d]
	LGD1069	-11.18	-87.77	-77.02
hRXRγ (Model)	ATRA	-9.40	-78.60	-83.12
	9cRA	-10.81	-83.76	-83.96
	Am80	nb[b]	nh[d]	nh[d]
	LGD1069	-10.98	-72.02	-78.99
hRARα (PDB ID: 3A9E)	ATRA	-11.52	-95.94	-101.01
	9cRA	nd[c]	-94.03	-94.41
	Am80	-11.12	-101.63	-103.84
	LGD1069	-9.16	-84.03	-97.24
hRARβ (PDB ID: 4DM8)	ATRA	-11.39	-107.72	-120.46
	9cRA	nd[c]	-112.03	-106.37
	Am80	-10.22	-122.27	-121.47
	LGD1069	-9.92	-89.47	-110.30
hRARγ (PDB ID: 2LBD)	ATRA	-14.13	-108.99	-117.13
	9cRA	-12.23	-103.19	-113.72
	Am80	nb[b]	nh[d]	nh[d]
	LGD1069	-9.35	-60.91	-87.14

[a] Ref. [1], [b] nb: does not bind, [c] nd: no data available, [d] nh: no hit

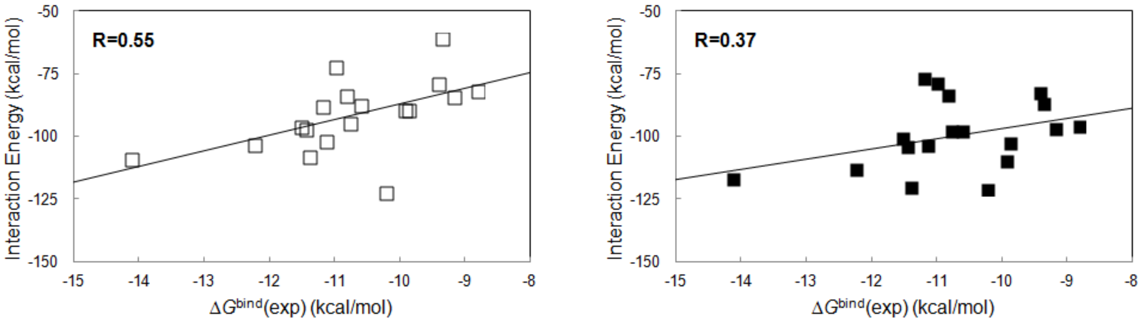

Figure S1. Correlations between ΔG^{bind}(exp) [1] and interaction energies of the most stable complex obtained from the docking simulations [2, 3] (left: the biomacromolecule-rigid and ligand-flexible conditions; right: the biomacromolecule- and ligand-flexible conditions) for the binding of the α, β, and γ subtypes of hRXR and hRAR LBDs with ATRA, 9cRA, Am80, and LGD1069.

Table S2. Receptor information used in this study [2].

Receptor	PDB ID	Ligand	Resolution [Å]	State
hRXRα	1FM9	9cRA	2.1	Heterodimer with hPPARγ LBD in agonist conformation
hRXRβ	1UHL	MEI[a]	2.9	Heterodimer with hLXRα LBD in agonist conformation
hRXRγ	–	9cRA	–	Model using hRXRα LBD (1FM9)[b]
hRARα	3A9E	ATRA	2.75	Heterodimer with mRXRα LBD in antagonist conformation
hRARβ	4DM8	9cRA	2.3	Homodimer
hRARγ	2LBD	ATRA	2.0	Monomer

[a] MEI: methoprenic acid, [b] for preparation, see Ref. [2]

References

1. Umemiya H, Fukasawa H, Ebisawa M, Eyrolles L, Kawachi E, Eisenmann G, Gronemeyer H, Hashimoto Y, Shudo K and Kagechika H (1997) Regulation of retinoidal actions by diazepinylbenzoic acids. Retinoid synergists which activate the RXR-RAR heterodimers. *J Med Chem* **40**, 4222−4234.

2. Tsuji M, Shudo K and Kagechika H (2015) Docking simulations suggest that all-*trans* retinoic acid could bind to retinoid X receptors. *J Comput Aided Mol Des* **29**, 975−988.

3. Tsuji M (2015) Docking Study with HyperChem, revision G1, Institute of Molecular Function, Saitama, Japan.
